# Implementation of the Perinatal Death Surveillance and Response guidelines: Lessons from annual health system strengthening interventions in the Rwenzori Sub‐Region, Western Uganda

**DOI:** 10.1002/nop2.524

**Published:** 2020-06-02

**Authors:** Enos Mirembe Masereka, Amelia Naturinda, Alex Tumusiime, Clement Munguiko

**Affiliations:** ^1^ Department of Nursing and Midwifery School of Medicine Kabale University Kabale Uganda; ^2^ Department of Nursing and Midwifery School of Health Sciences Mountains of the Moon University Fort Portal Uganda; ^3^ Infectious Diseases Institute School of Medicine College of Health Sciences Makerere University Kampala Uganda; ^4^ Department of Nursing School of Health Sciences Soroti University Soroti Uganda

**Keywords:** deaths, neonatal, perinatal, stillbirths, surveillance, Western Uganda

## Abstract

**Aim:**

To determine the health facility‐based perinatal mortality rate, its causes and avoidable factors using the perinatal mortality surveillance and response guidelines.

**Design:**

This was an action study conducted in one of the districts in Western Uganda from 1 January–31 December 2019.

**Methods:**

A total of 20 perinatal death cases were recruited consecutively. Data were collected using a Ministry of Health Perinatal Death Surveillance and Response (PDSR) questionnaire containing questions on pregnancy, delivery and immediate postnatal care. We used descriptive statistics to describe key data elements.

**Results:**

We found a health facility‐based perinatal mortality rate of 17.3 deaths per 1,000 live births. Birth asphyxia was the most common cause of perinatal deaths. Seven, three and ten mothers delayed seeking, reaching and receiving appropriate health care, respectively.

## 1 INTRODUCTION

Over the years, the global perinatal death rate has shown a slow decline despite the availability of systems and the that can change the trend (UNICEF & WHO, 2017). The Perinatal Death Surveillance and Response (PDSR) system is one possible model if well used. It integrates and builds on existing systems such as surveillance, health information and village health teams in Uganda (MOH, 2017). The PDSR system involves the identification of all perinatal deaths, timely notification and conducting a review to understand why the newborn died through consideration of medical, social and economic contributing factors. This is followed by the formulation of actions to prevent the occurence of similar deaths (MOH, 2017). Despite the available evidence that PDSR can generate solutions and contribute to the end of avoidable perinatal deaths, it is not widely used in many health facilities (Agaro et al., 2016). We determined the health facility‐based perinatal mortality rate, its causes and avoidable factors during a 1‐year rigorous PDSR guideline implementation in one of the districts in Western Uganda.

## 2 BACKGROUND

The global perinatal mortality rate continues to be high with about 37.4 deaths per 1,000 live births (De Bernis et al., 2016; UNICEF & WHO, 2017; Wang et al., 2016). Death is identified as a perinatal death if it occurred from 28 weeks of gestation to 7 days after birth. Perinatal deaths include stillbirths and early neonatal deaths. A total of 5.7 million perinatal deaths occur worldwide, of these 2.7 million are neonatal deaths and 2.6 million are stillbirths. A perinatal death is identified as a stillbirth if it occurred from 28 weeks of gestation and before delivery of the baby and as neonatal death if it occurred following the birth of the baby to seventh day after birth. Approximately 95% of the world perinatal deaths occur in South Asia and sub‐Saharan Africa (UNICEF & WHO, 2017; Wang et al., 2016). The occurence of stillbirths reflects the quality of antenatal care received by pregnant mothers, and the occurrence of neonatal deaths reflects the quality of newborn care provided during labour, childbirth and the immediate postnatal period (Nakimuli et al., 2015; Okonofua et al., 2019). In South Africa, about 50.4% of macerated stillbirths (MSB) and 50.7% of fresh stillbirths (FSB) were due to medical and obstetric conditions in pregnancy such as hypertension and obstetric haemorrhage. The main causes of Neonatal Deaths (NNDs) were immaturity (48.7%) and consequences of complicated birth such as birth hypoxia (40.6%) (Allanson, Muller, & Pattinson, 2015).

Although Uganda has registered a decline in infant and under‐five mortality from 158 in 2001 to 46.4 deaths per 1,000 live births in 2018, neonatal mortality has persistently remained at 27 deaths per 1,000 live births (MOH, 2013 & UNICEF, 2020). This has led to a slow reduction in overall under‐five mortality **(**Kananura et al., 2016 **).** Newborns continue to die due to the three delays; the delay to make a decision to seek, the delay to reach and the delay to receive appropriate obstetric and neonatal care at a health facility **(**Grady et al., 2017; Kananura et al., 2016). To address these delays, Uganda implements the Reproductive, Maternal, Newborn, Child and Adolescent Health (RMNCAH) sharpened plan that emphasizes implementation of key strategic shifts including reaching to the underserved areas and the high burden populations with high impact interventions, supporting women education, women empowerment, improving the economy and ensuring mutual accountability (MOH, 2013). Uganda implements the nine evidence‐based high impact interventions that include administering parenteral antibiotics in the treatment of obstetric infections, administering uterotonic drugs for active management of the third stage of labour and prevention of postpartum haemorrhage, use of parenteral anticonvulsants for the management of pre‐eclampsia and eclampsia, manual removal of placenta, removal of retained products of conception, conducting assisted vaginal delivery, performing neonatal resuscitation, performing caesarean delivery and administering blood transfusion (Bhutta et al., 2014; MOH, 2013).

Despite the roll‐out of these interventions, newborns continue to die and to understand why, health facilities are required to conduct PDSR (MOH, 2013). Despite the roll‐out of PDSR guidelines, they are rarely implemented in most health facilities in the country. For instance, in a study conducted in Oyam District in Uganda, only 34.8% of the health workers had ever participated in their implementation. Non‐existence of PDSR committees, the health workers being unaware of the PDSR process, lack of training of PDSR committee members, inadequate support supervision and lack of financial motivation of PDSR committee members were the factors associated with non‐functional PDSR system. In health facilities where PDSR committees existed, non‐attendance of review meetings by members, lack of knowledge of objectives of PDSR implementation, non‐implementation of committee recommendations and heavy workload of health workers were the challenges (Agaro et al., 2016). In the study settings, only 5% of the expected PDSR reports were submitted to the District Health Officer (DHO) and transmitted to the Ministry of Health in 2018. Due to this, a health system strengthening project incorporated interventions to support PDSR. In this action research, we present the health facility‐based perinatal mortality rate, its causes and avoidable factors from a vigorously implemented PDSR in Western Uganda.

## 3 THE STUDY

### 3.1 Design

This was an action study conducted as part of the key outputs of the authors. A midwife on duty in a health facility labour suit identified a perinatal death and notified the PDSR committee that conducted a review within 7 days. The study did not include perinatal deaths that did not have adequate clinical documentation and those that happened in homes. Following the review of each case, actions for improvement were generated and implemented. The study was conducted from 1 January–31 December 2019. At the inception, the study area had a total of nine health facilities; five of these had maternity settings where deliveries were conducted. Each maternity setting had a labour suite, antenatal and postnatal wings, with an average of two midwives. Midwives were trained in the basic emergency obstetric and newborn care skills and PDSR. Each labour suite had the minimum equipment required for safe and clean delivery such as delivery beds, delivery sets, assisted delivery equipment and newborn resuscitation supplies. The highest referral health facility in the area had a stand‐by ambulance and a well‐equipped theatre. Blood transfusion was one of the services offered at this health facility. Hard‐to‐reach villages with poor road network were supported with community tricycle ambulances to transport mothers seeking delivery services at night and those with danger signs. Each village had two Village Health Team (VHT) members trained in early identification of danger signs and referral. In 2019, the district had a total of 14,665 women in the reproductive age. About 3,630 pregnancies and 3,521 deliveries. A total of 2,084 of the deliveries took place in the health facilities (MOH, 2018). The Batuuku, Batooro and Bakonzo were the predominant tribes. The inhabitants participated in cattle keeping, crop growing, fishing along Lake Albert and engaged in small‐scale businesses as their key socio‐economic activities.

### 3.2 Sampling procedure

At the inception, PDSR committees were constituted in accordance with the PDSR guidelines per study health facility (MOH, 2017). Members such as the health facility in‐charge, maternity staff, laboratory representative, medical records representative, ambulance driver, medical stores representative, drug dispenser and VHT representative were included. The midwife in‐charge of maternity ward was the focal point person and the secretary of the committee, while the health facility in‐charge was the committee chairperson. Each death was listed by filling a form capturing the details of the death by the in‐charge maternity ward or a midwife on duty. The PDSR committee was notified of each identified death and each death was reviewed within seven days of notification (MOH, 2017). A total of 36 perinatal deaths occured during the entire year. Of these 20 were consectively recruited. A total of 16 perinatal deaths were excluded because of inadequate maternal and newborn clinical information.

### 3.3 Data collection and analysis procedures

**FIGURE 1:**
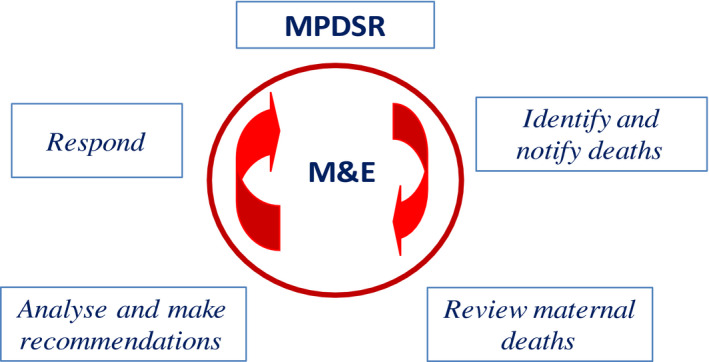
The continuous action cycle. M&E, monitoring and evaluation; MPDSR, Maternal, Perinatal, Death, Surveillance and Response

Data collection and analysis were guided by the PDSR guidelines and the continuous action cycle (MOH, 2017). In this system, perinatal death cases were identified and notified to key stakeholders, the death was reviewed, data were captured and analysed, and recommendations were suggested for improvement (Figure 1).

Following each death, a notification form was filled by the midwife on duty and copies were sent to the maternity ward in‐charge, health facility in‐charge, the district health officer and the Ministry of Health within 48 hr. Health facility death review committee thereafter reviewed the case within 7 days. During the review, data were collected using a pre‐tested and validated Ministry of Health Perinatal Death Review questionnaire (MOH, 2017). The questionnaire contained sections on demographic and obstetric information of the mother; care received during pregnancy, labour and immediate postpartum period, type of death, possible causes and associated factors. A death was identified as perinatal death if it occurred from 28 completed weeks of gestation up to 7 days after birth. A perinatal death was categorized as NND if it occurred during the first 7 days of life; a death was categorized as a stillbirth if it occurred before complete expulsion of the baby from its mother. Stillbirths were categorized as MSB if there were signs of body decomposition such as peeling off the skin by the time the infant was born and as FSB if the body was fresh by time of birth. Key data elements were filled in the questionnaire by the committee secretary after brainstorming, comparing and sharing of experiences among committee members. Ministry of Health guidelines were benchmarks for identifying deviations from normal in the care given to the mothers to arrive to the probable causes of perinatal death. The committee brainstormed possible factors associated with perinatal deaths and categorized them according to the three delays (the delay to make a decision to seek, the delay to reach and the delay to receive appropriate care). Key data elements such as obstetric histories, antenatal, labour and the immediate postnatal care, and avoidable factors were summarized using descriptive statistics.

### 3.4 Ethical considerations

The protocol was approved by a local ethics committee at Mountains of the Moon University. The PDSR committee members adhered to the PDSR ethical guidelines that emphasize no naming and no blaming and signed a confidentiality agreement form. While referring to the deceased baby and the mother during committee meetings, only initials were used.

## 4 RESULTS

### 4.1 Maternal socio‐demographic and obstetric histories

Fifteen mothers were aged 18–35 years, sixteen had fewer than four children, seventeen had no history of abortion and twenty carried singleton pregnancies (Table 1).

**TABLE 1:** Maternal socio‐demographic and obstetric histories

Variable	Frequency *N* = 20	Percentage
Age		
<18	4	20
18–35	15	75
>35	1	5
Parity		
≤4	16	80
>4	4	20
History of abortion		
Yes	3	15
No	17	85
Type of pregnancy		
Singleton	20	100
Multiple	0	0
No. of living children		
≤4	17	85
>4	3	15

### 4.2 Health Facility‐Based Perinatal Mortality Rate

During the study period, a total of 2,084 deliveries were conducted and 36 perinatal deaths were registered, causing a health facility‐based perinatal mortality rate of 17.3 deaths per 1,000 live births.

### 4.3 Antenatal care and medical risks diagnosed during pregnancy

Thirteen mothers attended less than 4 antenatal visits, eleven mothers received less than two doses of intermittent presumptive treatment (IPTp) for malaria in pregnancy and fourteen mothers received less than two doses of tetanus and diphtheria (TD) vaccine. The other services such as HIV testing, syphilis testing, iron and folic acid supplementation and deworming were well provided during antenatal care. Severe malaria (four mothers), urinary tract infections (four mothers) and being young primigravida (four mothers) were the most common medical and obstetric risks identified during pregnancy (Table 2).

**TABLE 2:** Antenatal care and medical risks diagnosed during pregnancy

Variable	Frequency *N* = 20	Percentage
Antenatal care		
No. of ANC		
≥4	7	35
<4	13	65
No. of IPTp doses		
≥2	9	45
<2	11	55
No. of TD doses		
≥2	6	30
<2	14	70
HIV testing		
Yes	19	95
No	1	5
HIV status		
Positive	2	10
Negative	18	90
Syphilis testing		
Yes	18	90
No	2	10
Syphilis status		
Positive	1	5
Negative	19	95
Iron and folic acid supplementation		
Yes	18	90
No	2	10
Deworming status		
Up to date	18	90
Not up to date	2	10
Medical risks diagnosed in pregnancy		
Severe malaria	4	20
Urinary tract infections (UTI)	4	20
Young prime gravida	4	20
No risk	3	15
Pre‐Eclampsia	1	5
Hypertension	1	5
Diabetes mellitus	1	5
Multiparity	1	5
pPROM	1	5
Anaemia	1	5
HIV	1	5
Threatened abortion	1	5
HIV	1	5
Syphilis	1	5

Abbreviations: ANC, Antenatal care; HIV, Human Immunodeficiency Syndrome; IPTp, Intermittent Presumptive Treatment for Malaria in pregnancy; pPROM, Premature Prelabour Rupture of Membranes; TD, Tetanus & Deptheria.

### 4.4 Labour progress and obstetric risks

Twelve mothers were at term, delivery took place in the health facilities in ninteen mothers, eighteen had spontaneous vaginal delivery, and twelve babies had no heart function by birth. Breech delivery (two mothers), premature pre‐labour rupture of membranes (three mothers) and obstructed labour (two mothers) (Table 3).

**TABLE 3:** Labour progress and obstetric risks

Variable	Frequency *N* = 20	Percentage
Labour progress		
Weeks of gestation at birth		
37–42	12	60
<37	8	40
Place of delivery		
Health facility	19	95
Home	1	5
Foetal heart at birth		
Present	8	40
Absent	12	60
Mode of delivery		
Spontaneous vaginal	18	90
Caesarian section	2	10
Correct use of partograph (*n*_o_ = 8)		
Yes	7	87.5
No	1	12.5
Time elapsed between decision for caesarian section (*n*_o_ =2)		
<30 min	1	50
≥30 min	1	50
Risks diagnosed during labour and delivery		
PROM	3	15
Breech delivery	2	10
Obstructed labour	2	10
Prematurity	1	5
Short cord	1	5
Placenta abruptio	1	5
Ruptured uterus	1	5

Abbreviation: PROM, Premature Rupture of Membranes.

### 4.5 Condition of the baby after birth and probable causes of perinatal deaths

Twelve babies weighed more than 2.5 kg and twelve were lifeless (with Apgar score zero) at birth. Eight babies were asphyxiated, and all were resuscitated despite the outcome. Eightt perinatal deaths were neonatal deaths, seven were macerated stillbirths and five were fresh stillbirths. Birth asphyxia (eight babies), malaria in pregnancy (four babies) and urinary tract infections (four babies) were the most common probable causes of perinatal deaths. Birth asphyxia in the PDSR meetings was thought to be caused by pre‐existing infections in pregnancy, prolonged labour, obstructed labour, malaria and anaemia in pregnancy, placenta abruption, rupture of the uterus, premature rupture of membranes and unsuccessful breech extraction (Table 4).

**TABLE 4:** Condition of the baby after birth and causes of perinatal deaths

Variable	Frequency *N* = 20	Percentage
Condition of the baby after birth		
Apgar		
0 (No life)	12	60
1–3 (Severe birth asphyxia)	4	20
4–6 (moderate birth asphyxia)	2	10
7 ≥ 9 (mild birth asphyxia)	2	10
Baby resuscitation (*n*_o_ = 8)		
Suction	5	62.5
Bag and mask	3	37.5
Baby’s weight at birth		
Normal (>2.5 kg)	12	60
Low birth weight (1.5–2.5 kg)	6	30
Very low Birth weight (1–1.5 kg)	2	10
Extremely low birth weight (<1 kg)	0	0
Sex		
Male	12	60
Female	8	40
Type of perinatal death		
Macerated still births (MSBs)	7	35
Fresh still births (FSBs)	5	25
Neonatal deaths (NNDs)	8	40
Probable causes of perinatal deaths		
MSBs		
Malaria in pregnancy	3	15
UTI in pregnancy	3	15
Unexplained MSB	1	5
FSBs		
Prematurity	2	10
Prolonged labour	1	5
Hypoglyceamia	1	5
Congenital abnormalities	1	5
NNDs		
Birth asphyxia	8	40

### 4.6 Avoidable factors for perinatal deaths

These were the factors contributing to the delays to seeking, reaching and receiving appropriate health interventions. Seven mothers delayed to make a decision to seek health care, three mothers delayed reaching the health facility and ten mothers delayed receiving appropriate interventions at the health facility. During the PDSR committee meetings, the delay to make aa decision to seek health care was thought to be due to stigma associated with being young primegravida, lack of partner involvement, the failure to recognize maternal and foetal danger signs early in pregnancy. The delay to reach the health facility was linked to long distances between the health facilities and households and the lack of means of transport. The delay to receive appropriate obstetric care at the health facilities was thought to be due to failure of midwives to diagnose complications early in pregnancy, limited breech delivery skills, the lack of paediatric High Dependence Unit (HDU) equipment, the absence of anaesthetic personnel, communication gap between midwives and aphasic mother and the lack of sign language skills (Table 5).

**TABLE 5:** Causes of perinatal deaths linked with respective avoidable factors and actions

Case No.	Cause of perinatal death	Avoidable factor	Type of delay
1	Severe malaria in pregnancy	Long distance to the nearby health facility	Delay to reach the health facility
2	Severe malaria in pregnancy	Failure to recognize danger signs in pregnancy	Delay to seek health care
3	Urinary Tract Infections (UTI) in pregnancy	Long distance to the nearby health facility	Delay to reach the health facility
4	Severe malaria in pregnancy	Stock outs of antimalarial drugs & mother was unable to buy	Delay to provide appropriate care
5	Prolonged second stage of labour	Limited instrumental delivery skills	Delay to provide appropriate care
6	Gross congenital abnormalities	Lack of USS services in antenatal care	Delay to provide appropriate care
7	Complications of prematurity	Failure to communicate with aphasic client during ANC	Delay to provide appropriate care
8	Birth Asphyxia due to infections in pregnancy	Failure to recognize danger signs in pregnancy	Delay to seek health care
9	Birth asphyxia due to ruptured uterus	Long distance to the health facility	Delay to reach the health facility
10	Birth asphyxia due to pPROM and oligohydromnios	Failure to recognize danger signs in pregnancy	Delay to seek health care
11	Hypoglycaemia due to maternal diabetes mellitus	Failure to feed the child immediately after birth	Delay to provide appropriate care
12	Birth asphyxia due to placenta abruption	Failure to diagnose short cord in pregnancy	Delay to provide appropriate care
13	Birth asphyxia due to obstructed labour	Attended few antenatal care visits	Delay to provide appropriate care
14	UTI in pregnancy	Started antenatal care too late	Delay to seek health care
15	Complications of prematurity	Lack of HDU equipment	Delay to provide appropriate care
16	UTI in pregnancy	Failure to recognize danger signs in pregnancy	Delay to seek health care
17	Unexplained macerated still birth	Failure to recognize danger signs in pregnancy	Delay to seek health care
18	Birth asphyxia due to obstructed labour	Failure to diagnose obstructed labour	Delay to provide appropriate care
19	Birth asphyxia due to prolonged breech extraction	Limited breech delivery & extraction skills	Delay to provide appropriate care
20	Asphyxia due to pre‐existing anaemia in pregnancy	Never attended ANC due to stigma of being young PG	Delay to seek health care

Abbreviations: ANC, Antenatal Care; HDU, High Dependency Unit; pPROM, Premature Prelabour Rupture of Membranes; USS, Ultra Sound Scan; UTI, Urinary Tract Infections.

## 5 DISCUSSIONS

### 5.1 Health facility‐based perinatal mortality rate

In this action study, we found a health facility‐based perinatal mortality rate of 17.3 deaths per 1,000 live births. This is more likely to be higher than estimated since 40.8% of the deliveries happen at home (MOH, 2018). Home perinatal deaths are never reported in the health management and information system due to poor community reporting system in Uganda (MOH, 2018). Determination of the consolidated perinatal mortality rate therefore requires a community‐based study. Nonetheless, to achieve the sustainable development goals (SDGs) and targets, preventable perinatal deaths in health facilities must come to an end (MOH, 2017). To achieve this, health facilities should provide quality care to pregnant mothers during pregnancy, childbirth and in the postnatal period (MOH, 2013).

### 5.2 Antenatal care and medical risks diagnosed during pregnancy

In this study, thirteen mothers attended less than four antenatal visits. According to the Ministry of Health, four visits is the minimum number any mother should attend during pregnancy. Attending fewer visits could be attributed to the lack of means of transport amidst long distannces to reach nearby health facility and the limited integrated outreaches that would taake services closer to pregnant women. Attending fewere visits is more likely to decrease the probability of identifying foetuses at risk of foetal death (Vogel et al., 2013). More so, mothers end up missing some preventive antenatal care packages as there are prolonged gaps between antenatal visits (Vogel et al., 2013). For instance, in this study, it was found that most mothers received less than two doses of IPTp and TD despite most delivery at term. Receiving preventive medicines during antenatal care has been linked with reduced perinatal mortality rate(Andargie, Berhane, Worku, & Kebede, 2013; Menéndez et al., 2010). On the other hand, it has been found that attending more antenatal visits may not necessarily guarantee improved quality of care in the event that supplies may be out of stock and critical human resource may not be available (Vogel et al., 2013).

In this study, the most prevalent risk factors during pregnancy were severe malaria (four mothers) and urinary tract infections (four mothers). Studies have found perinatal infections as cause of perinatal mortality. Perinatal infections cause substantial perinatal mortality (Adam, Elhassan, Abd Elrahium, Ali, & Adam, 2011; Valea et al., 2012). It was also discovered that four mothers in this study were primigravida, and similarly, primigravidity has been linked with perinatal mortality in some studies. This could be due to the fact that a first‐time mother may lack key information about pregnancy care and the first pregnancy may be her first learning experience (Aslam et al., 2014). In our study, we do not underrate the fact that a three pregnant mothers did not have detectable danger signs. Like other studies, neonatal deaths were more likely to have a healthy mother. This has been found very challenging, given that the ability to predict these poor outcomes during antenatal by assessment of the mother is lost and making prenatal deaths unpredictable. This calls for midwives to be ready and have skills to reverse asphyxia whenever it happens in any mother (Allanson et al., 2015; Gordon, Raynes‐Greenow, McGeechan, Morris, & Jeffery, 2013).

### 5.3 Labour progress and obstetric risks

During labour, eighteen mothers delivered by spontaneous vaginal delivery. Twelve babies had no heartbeat after birth despite monitoring every labour where foetal heart beat was present using a partograph. However, monitoring with a partograph does not necessarily guarantee good labour outcome; other factors must be considered such as the quality of the partograph determines the extent to which information recorded reflects the maternal and foetal conditions (Ashish, Wrammert, Clark, Ewald, & Målqvist, 2016; Lavender, Hart, & Smyth, 2013). The skills of the midwife on how to capture maternal and foetal conditions will determine the quality of the partograph (Ashish et al., 2016; Lavender et al., 2013). A well‐documented partograph should help detect obstetric complications emerging during labour, elicit action when there is a detectable deviation from normal of maternal or foetal condition and should help to reduce the risk of perinatal mortality (Ashish et al., 2016). The most commonly found obstetric risks in this study were breech delivery, obstructed labour and premature rupture of membrane. Studies have linked unsuccessful breech extraction, prolonged labour (Aslam et al., 2014), obstructed labour (Mengesha & Sahle, 2017; Mmbaga et al., 2012), and premature rupture of membranes (Gezer et al., 2013; Margato, Martins, Júnior, & Nomura, 2012) with perinatal mortality entirely because of limitted skills, limitted human resources, lack of required equipment, medical supplies and drugs.

### 5.4 Causes of perinatal deaths

Due to medical and obstetric complications that presented during pregnancy, labour and childbirth established in this study, most babies died of birth asphyxia in the first seven days of birth. this is similar to findings in other studies where birth asphyxia was the leading cause of perinatal deaths (Aslam et al., 2014; Mengesha & Sahle, 2017; Mmbaga et al., 2012). Deahtshappening in the immediate post partum period categorized as neonatal deaths and those happening during labour and slightly before categorized as FSBs reflect the quality of care provided during labour and childbirth (Mengesha & Sahle, 2017). This implies that improving care during labour and immediate postpartum period would cut down the perinatal mortality rate by a considerable proportion. However, in this study, eight perinatal deaths were macerated stillbirths, which reflects the quality of antenatal care (Mengesha & Sahle, 2017); this finding is consistent with the fact that thirteen pregnant mothers attended less than four antenatal visits and could have missed packages of interventions to prevent common medical complications during pregnancy. The second most common causes of death were severe malaria and urinary tract infections and the third was prematurity. These conditions turn to be a big problem because mothers start antenatal care too late, failure to recognize danger signs in pregancy, failure to seek early and emergency obstetric care, mismanagement due to stockout of drugs as narrated in the PDSR meetings. Prematurity can be caused by most of the conditions occurring in pregnancy including pre‐eclampsia and eclampsia, malaria and urinary tract infections. Premature babies have no capacity to sustain extrauterine life and need thermoregulatory, respiratory and nutritional support. If settings cannot afford related care and support, life is lost (Allanson et al., 2015; Aslam et al., 2014; Mengesha & Sahle, 2017; Ota et al., 2014).

### 5.5 Avoidable factors for perinatal deaths

Seven mothers delayed to seek health care; in the PDSR meetings, it was discovered that some mothers delayed to seek health care due to stigma associated with being young primigravida (15 years), lack of partner involvement and failure to recognize maternal and foetal danger signs early in pregnancy. This is like other studies where few women knew three or more danger signs; early recognition of danger signs is more likely to prompt the mother to seek early care and prevent foetal death (Pembe et al., 2009). In other studies, the delays were due to underestimation of the severity of the complication and bad experience with the healthcare system (Getiye & Fantahun, 2017; Merali et al., 2014; Musafili et al., 2017). Three mothers delayed reaching a health facility due to long distances as one of the challenges. In other studies, delay in reaching an appropriate medical facility was due to lack of transportation and seeking care at more than one medical facility (Getiye & Fantahun, 2017; Musafili et al., 2017; Ota et al., 2014).

Ten mothers delayed to receive care at the health facility; the delay to receive appropriate interventions was due to failure to diagnose complications early in pregnancy, lack of critical human resource such as anaesthetic officer, lack of resuscitation equipment and communication gap as some mothers were aphasic and could not talk and the midwives lack sign language skills to understand or be understood by the mothers. Failure to diagnose complications early in pregnancy was due to the lack of critical equipment such as an ultrasound scan, which makes possible the diagnosis of conditions such as short cord and congenital abnormalities that are particularly implicated in pathogenesis of asphyxia in some of the cases. Similar to other studies, shortage of medical supplies, lack of equipment, lack of trained personnel and incompetence of the available staff cause delay to receive appropriate care (Getiye & Fantahun, 2017; Musafili et al., 2017; Ota et al., 2014).

## 6 CONCLUSION

A total of 20 perinatal deaths were reviewed, and we found a perinatal mortality rate of 17.3 deaths per 1,000 live births. Of the twenty perinatal deaths, eight were neonatal deaths, seven were macerated and five were fresh stillbirths. Seven mothers of the babies who died delayed to seek health care due to stigma associated with being young primigravida, lack of partner involvement and failure to recognize maternal and foetal danger signs early in pregnancy, Ten mothers delayed receiving appropriate care due to failure to diagnose complications early in pregnancy, lack of critical human resource such as anaesthetic officer, lack of resuscitation equipment and communication gap as some mothers were aphasic, and three mothers delayed to reach a health facility due to long distances and lack of means of transport.

## 7 RECOMMENDATIONS

We recommend interventions such as health educating pregnant women on danger signs and use of mosquito nets in pregnancy, health educating pregnant women on early attendance to care during pregnancy and labour, strengthening continuous medical education on pelvic assessments, communicating expected date of delivery for mothers to VHTs for appropriate follow‐up and referral to health facility, procuring and supplying ultrasound scan machines to all health facilities conducting antenatal care, training midwives in basics of obstetric ultrasonography and sign language.

## 8 STUDY LIMITATIONS

1.Home perinatal deaths were not included in the study due to lack of clear reporting system at community level and skepticism on obtaining mothers' detailed clinical information

2. Sixteen health facility perinatal deaths were not reviewed because of difficulty obtaining detailed clinical information. The PDSR committees tried to exhaustiveely present and discuss all other perinatal deaths with well documented clinical information

## CONFLICT OF INTEREST

The authors declare no competing interests in this study.

## AUTHOR CONTRIBUTIONS

Enos Mirembe Masereka, Amelia Naturinda and Alex Tumusiime conceived this study, and collected and analysed the data. Enos Masereka Mirembe and Clement Munguiko wrote the manuscript.
